# A Treatise on the Diseases of the Nervous System

**Published:** 1891-08

**Authors:** 


A Treatise on the Diseases of the Nervous System. By William A. Hammond, M. D., Surgeon-General U. S. Army (Retired List); late Professor of Diseases of the Mind and Nervous System in the College of Physicians and Surgeons of New York, the Bellevue Hospital Medical College, the University of the City of New York, and the New York Post-Graduate Medical School and Hospital, etc. With the collaboration of Græme M. Hammond, M. D., Professor of Diseases of the Mind and Nervous System in the New York Post-Graduate Medical School and Hospital, etc. With 118 illustrations. Ninth edition, with corrections and additions. 8vo, pp.—932. New York: D. Appleton & Company. 1891. Buffalo: Otto Ulbrich. Price, cloth, $5.00.

A book which has passed through eight large editions, needs no introduction to the medical profession, no words of praise, and hardly any adverse criticism; the medical public has long ere this reviewed its pages and noted the points of strength and of weakness. This ninth edition has been carefully revised and brought up to the present time by the author's son, Dr. Græme M. Hammond, and consequently stands in the front rank of text-books in neurology.

The Appletons, as is their usual custom, have done their part of the work admirably and deserve no small amount of praise.

